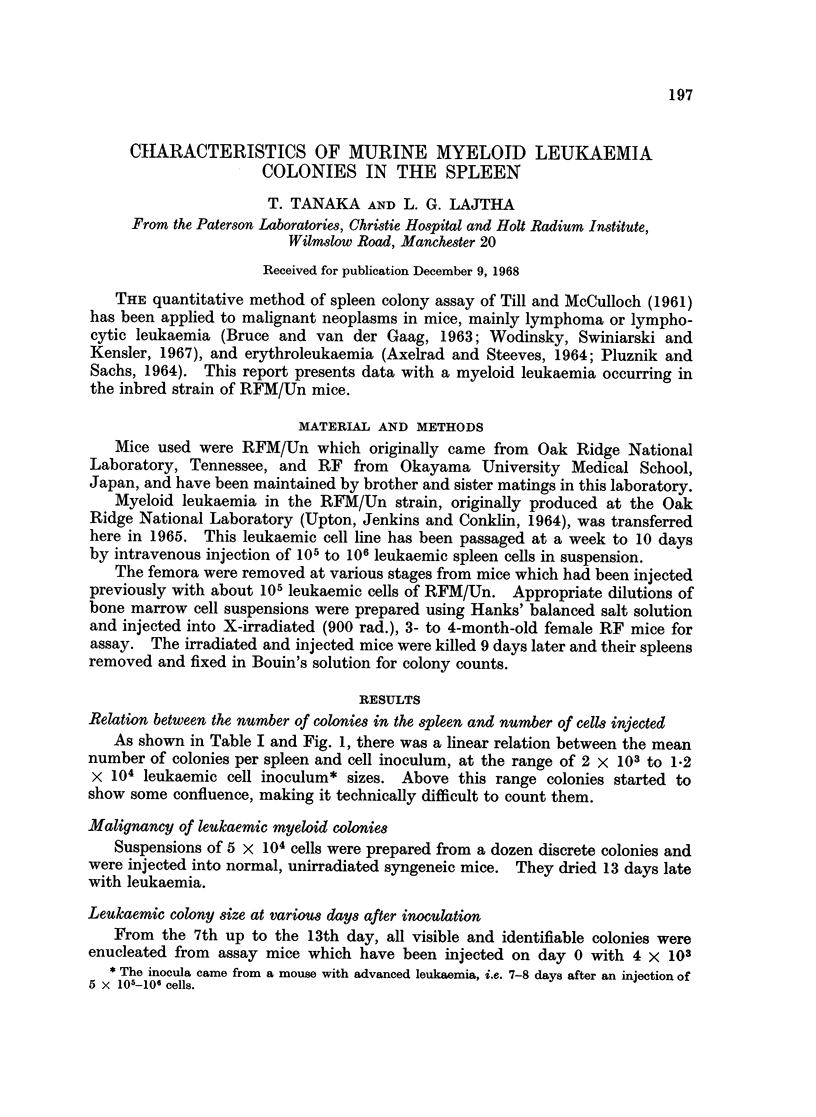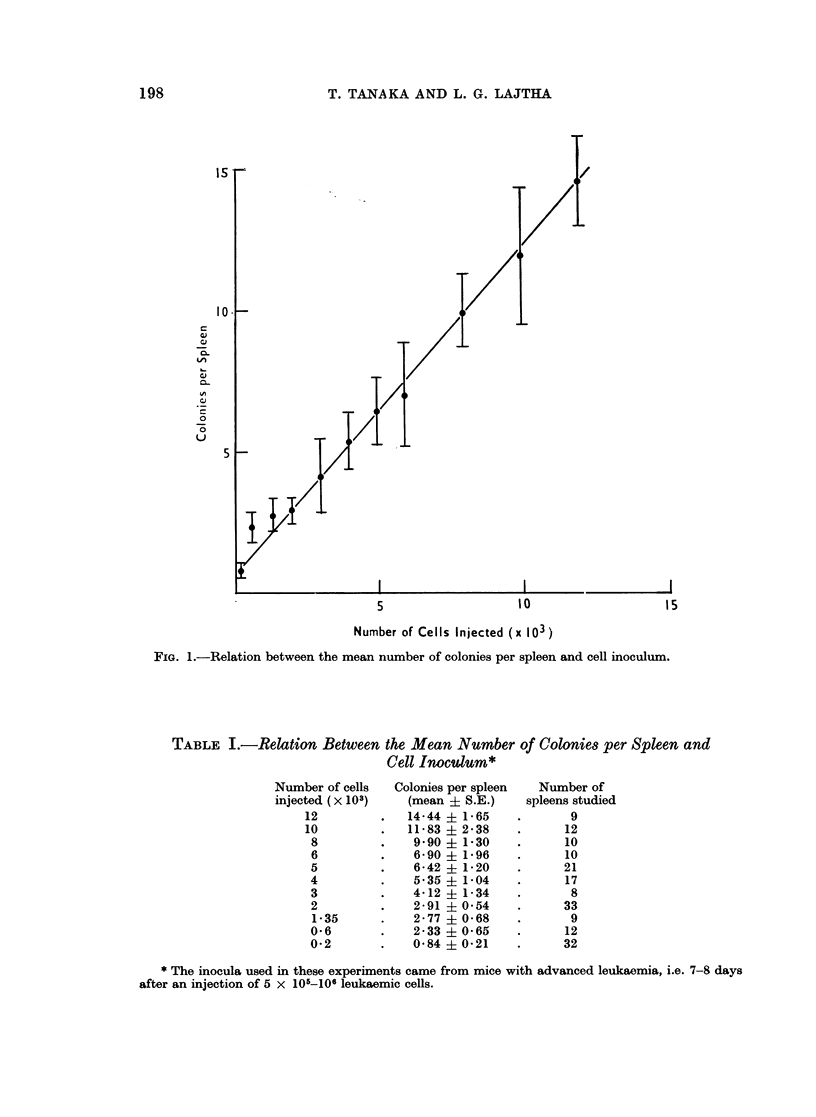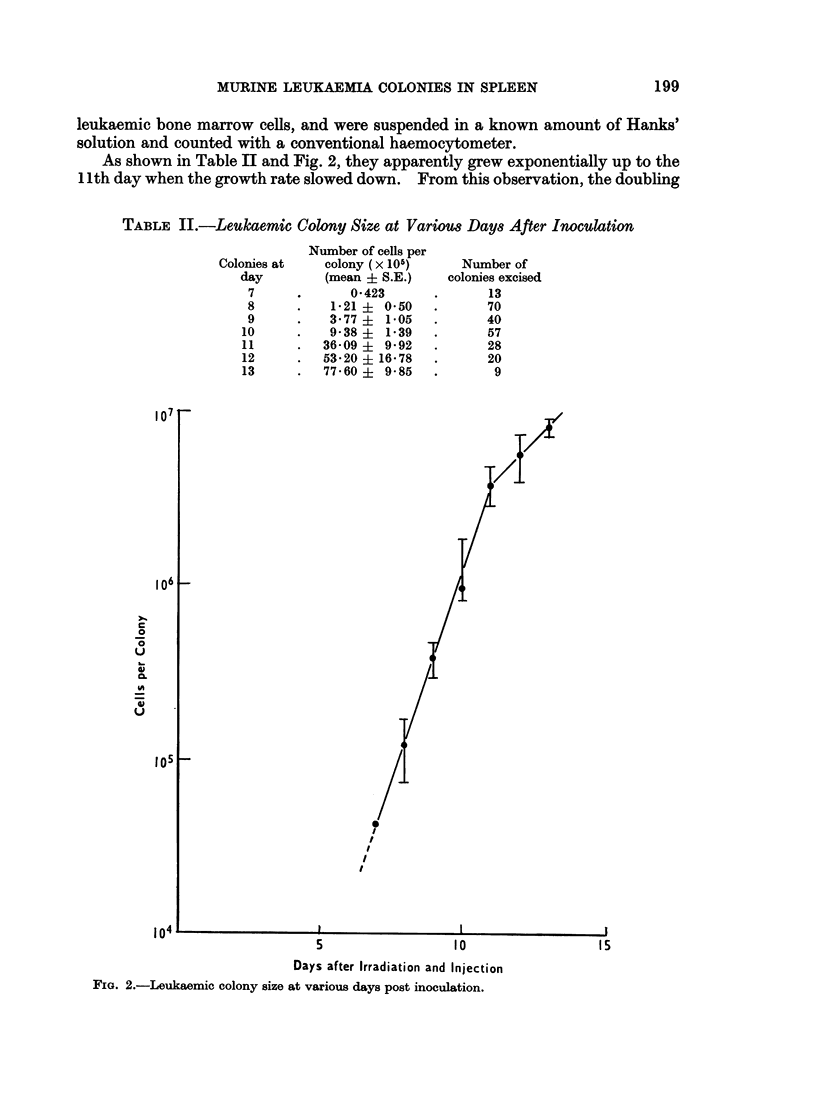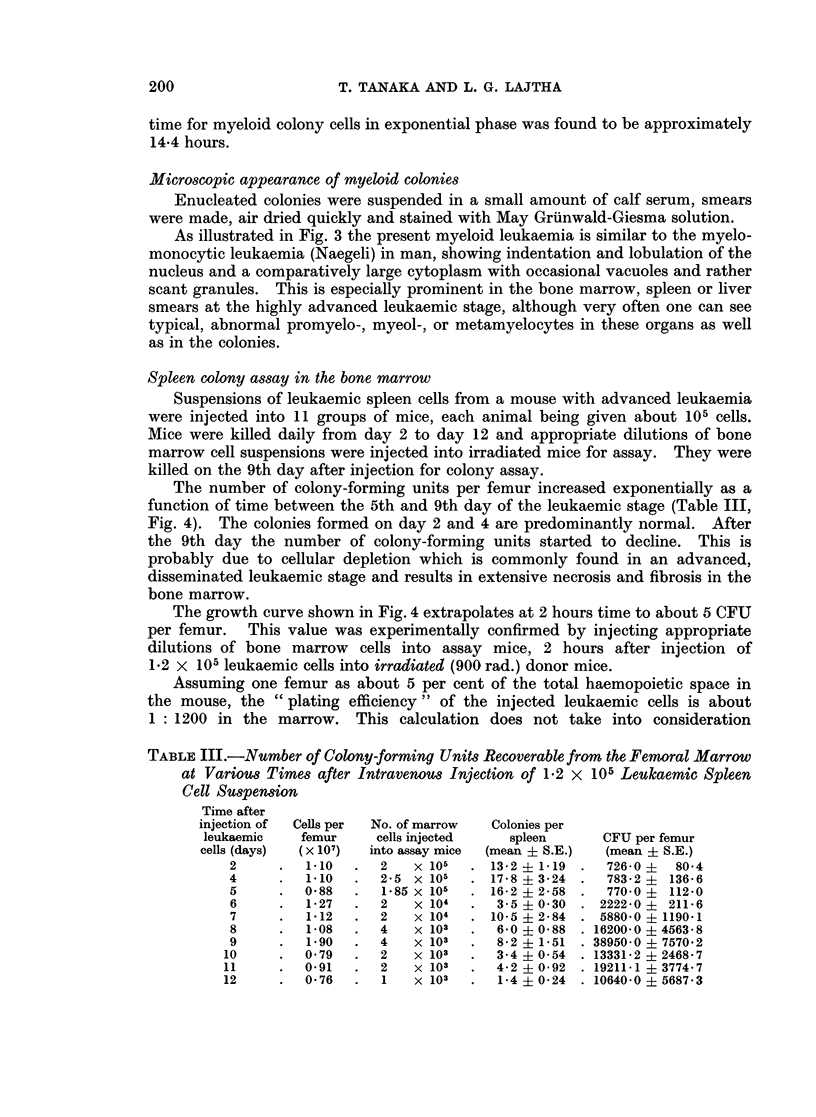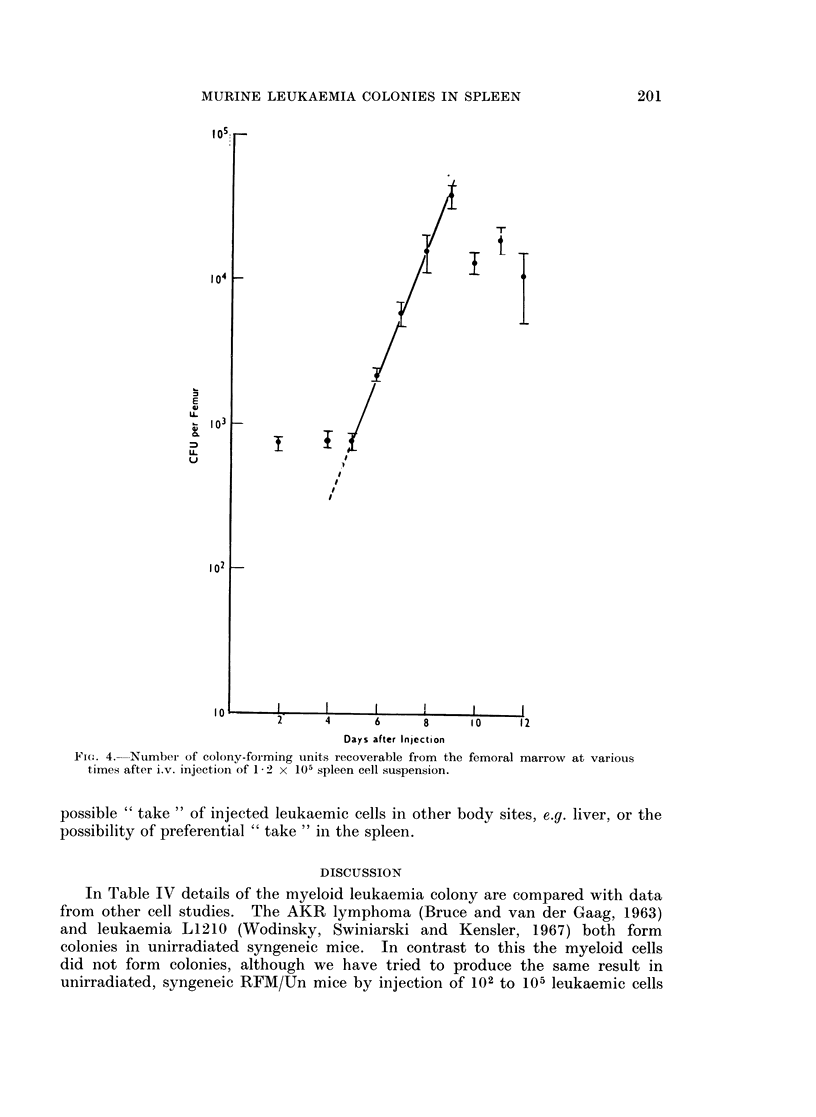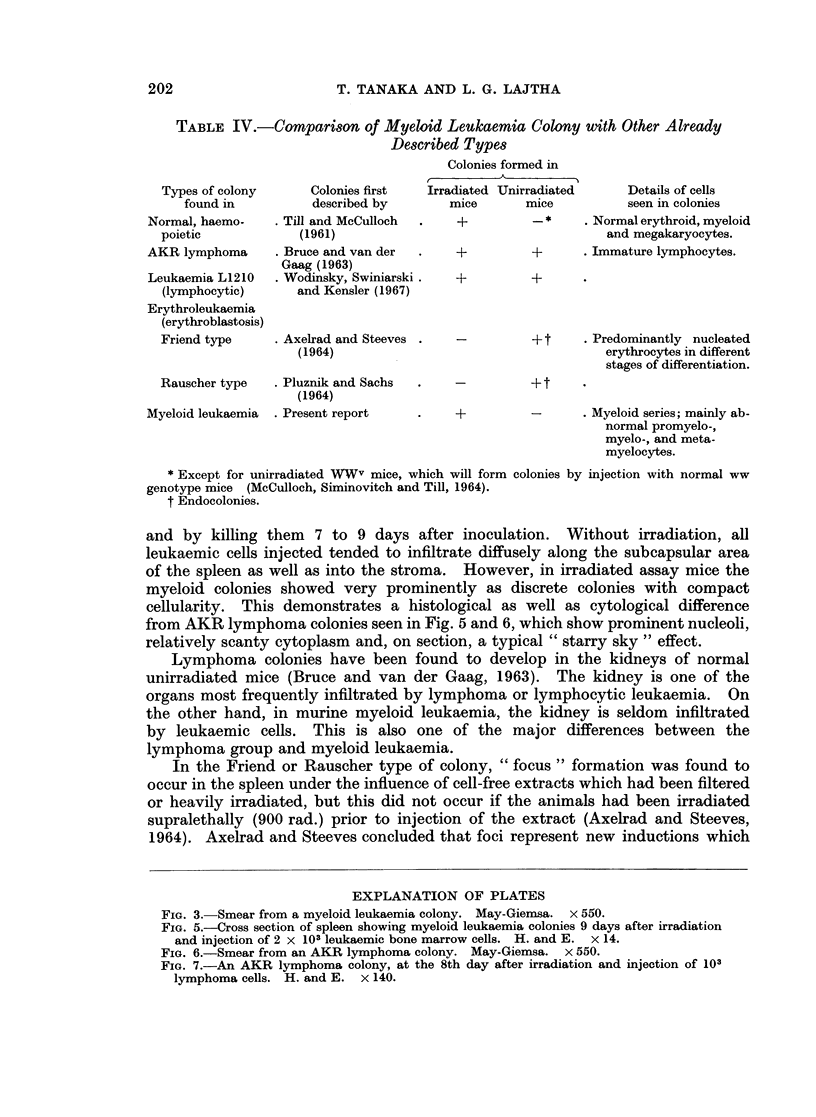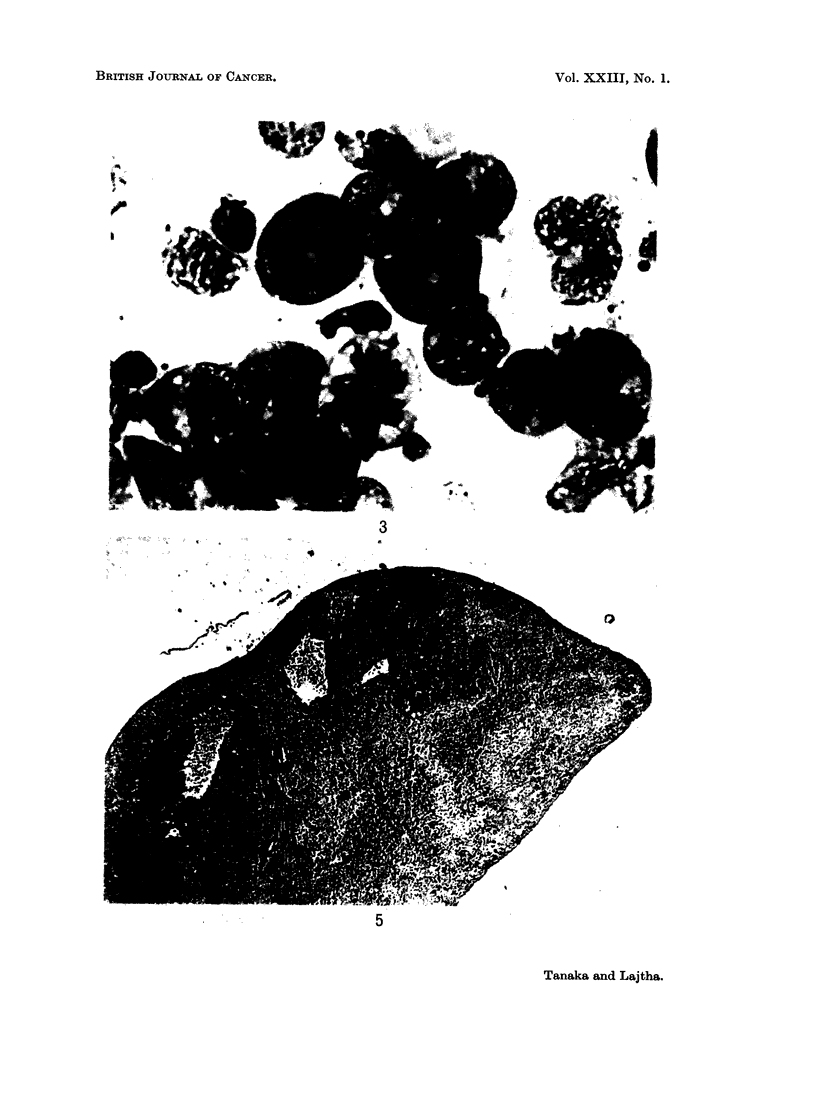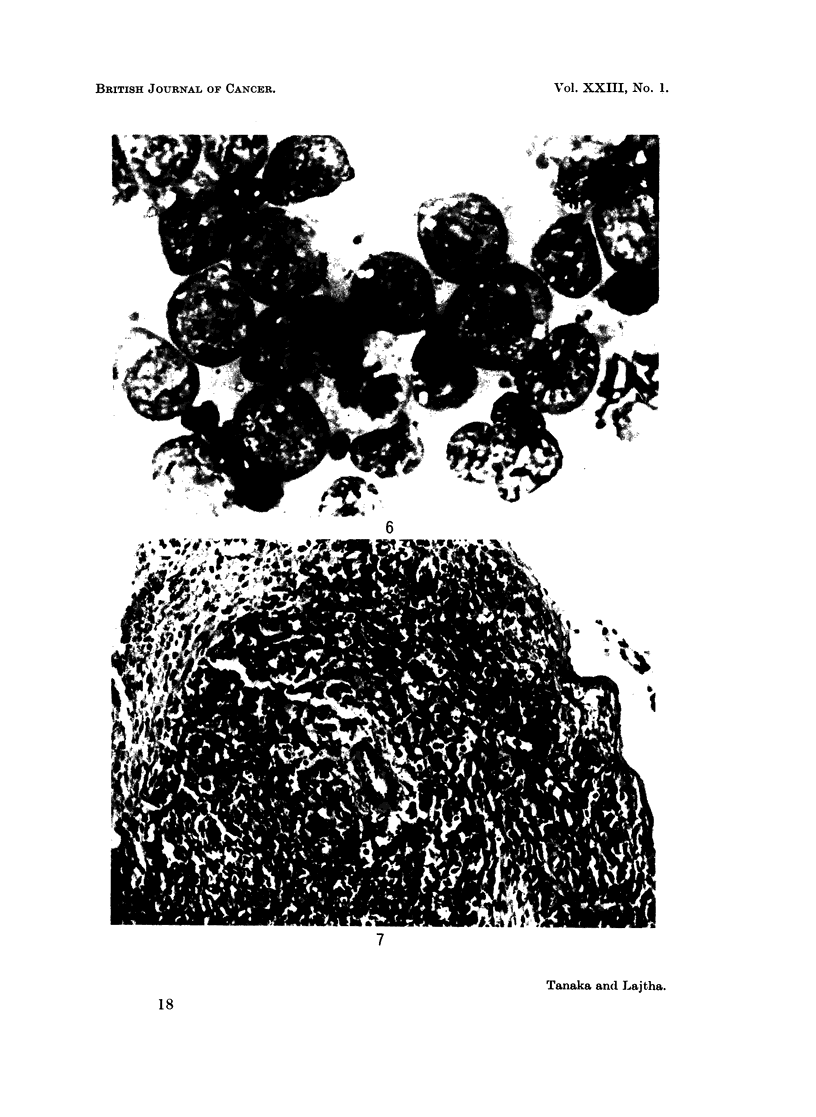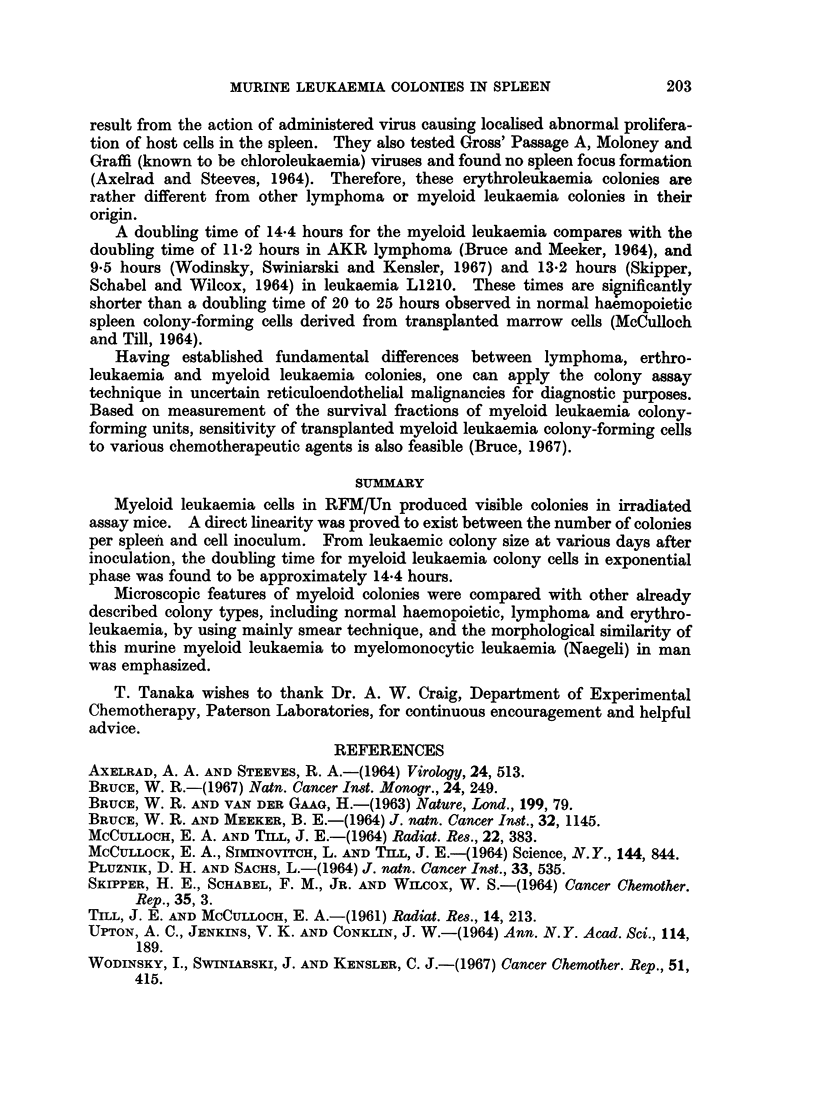# Characteristics of murine myelokd leukaemia colonies in the spleen.

**DOI:** 10.1038/bjc.1969.28

**Published:** 1969-03

**Authors:** T. Tanaka, L. G. Lajtha

## Abstract

**Images:**


### CHARACTERISTICS OF MURINE MYELOID LEUKAEMIA COLONIES IN THE SPLEEN

T. TANAKA AND L. G. LAJTHA

*From the Paterson Laboratories, Christie Hospital and Holt Radium Institute, Wilmslow Road, Manchester 20*

Received for publication December 9, 1968

THE quantitative method of spleen colony assay of Till and McCulloch (1961) has been applied to malignant neoplasms in mice, mainly lymphoma or lymphocytic leukaemia (Bruce and van der Gaag, 1963; Wodinsky, Swiniarski and Kensler, 1967), and erythroleukaemia (Axelrad and Steeves, 1964; Pluznik and Sachs, 1964). This report presents data with a myeloid leukaemia occurring in the inbred strain of RFM/Un mice.

### MATERIAL AND METHODS

Mice used were RFM/Un which originally came from Oak Ridge National Laboratory, Tennessee, and RF from Okayama University Medical School, Japan, and have been maintained by brother and sister matings in this laboratory.

Myeloid leukaemia in the RFM/Un strain, originally produced at the Oak Ridge National Laboratory (Upton, Jenkins and Conklin, 1964), was transferred here in 1965. This leukaemic cell line has been passaged at a week to 10 days by intravenous injection of $10^5$ to $10^6$ leukaemic spleen cells in suspension.

The femora were removed at various stages from mice which had been injected previously with about $10^5$ leukaemic cells of RFM/Un. Appropriate dilutions of bone marrow cell suspensions were prepared using Hanks' balanced salt solution and injected into X-irradiated (900 rad.), 3- to 4-month-old female RF mice for assay. The irradiated and injected mice were killed 9 days later and their spleens removed and fixed in Bouin's solution for colony counts.

### RESULTS

### *Relation between the number of colonies in the spleen and number of cells injected*

As shown in Table I and Fig. 1, there was a linear relation between the mean number of colonies per spleen and cell inoculum, at the range of $2 \times 10^3$ to $1{\cdot}2 \times 10^4$ leukaemic cell inoculum* sizes. Above this range colonies started to show some confluence, making it technically difficult to count them.

### *Malignancy of leukaemic myeloid colonies*

Suspensions of $5 \times 10^4$ cells were prepared from a dozen discrete colonies and were injected into normal, unirradiated syngeneic mice. They dried 13 days late with leukaemia.

### *Leukaemic colony size at various days after inoculation*

From the 7th up to the 13th day, all visible and identifiable colonies were enucleated from assay mice which have been injected on day 0 with $4 \times 10^3$

* The inocula came from a mouse with advanced leukaemia, *i.e.* 7–8 days after an injection of $5 \times 10^5$–$10^6$ cells.

FIG. 1.—Relation between the mean number of colonies per spleen and cell inoculum.

TABLE I.—*Relation Between the Mean Number of Colonies per Spleen and Cell Inoculum**

| Number of cells injected ( $\times 10^3$) | Colonies per spleen (mean $\pm$ S.E.) | Number of spleens studied |
|---|---|---|
| 12 | 14·44 $\pm$ 1·65 | 9 |
| 10 | 11·83 $\pm$ 2·38 | 12 |
| 8 | 9·90 $\pm$ 1·30 | 10 |
| 6 | 6·90 $\pm$ 1·96 | 10 |
| 5 | 6·42 $\pm$ 1·20 | 21 |
| 4 | 5·35 $\pm$ 1·04 | 17 |
| 3 | 4·12 $\pm$ 1·34 | 8 |
| 2 | 2·91 $\pm$ 0·54 | 33 |
| 1·35 | 2·77 $\pm$ 0·68 | 9 |
| 0·6 | 2·33 $\pm$ 0·65 | 12 |
| 0·2 | 0·84 $\pm$ 0·21 | 32 |

* The inocula used in these experiments came from mice with advanced leukaemia, i.e. 7–8 days after an injection of 5 $\times$ $10^5$–$10^6$ leukaemic cells.

leukaemic bone marrow cells, and were suspended in a known amount of Hanks' solution and counted with a conventional haemocytometer.

As shown in Table II and Fig. 2, they apparently grew exponentially up to the 11th day when the growth rate slowed down. From this observation, the doubling

TABLE II.—*Leukaemic Colony Size at Various Days After Inoculation*

| Colonies at day | Number of cells per colony ($\times 10^5$) (mean $\pm$ S.E.) | Number of colonies excised |
|---|---|---|
| 7 | 0·423 | 13 |
| 8 | 1·21 ± 0·50 | 70 |
| 9 | 3·77 ± 1·05 | 40 |
| 10 | 9·38 ± 1·39 | 57 |
| 11 | 36·09 ± 9·92 | 28 |
| 12 | 53·20 ± 16·78 | 20 |
| 13 | 77·60 ± 9·85 | 9 |

FIG. 2.—Leukaemic colony size at various days post inoculation.

time for myeloid colony cells in exponential phase was found to be approximately 14·4 hours.

*Microscopic appearance of myeloid colonies*

Enucleated colonies were suspended in a small amount of calf serum, smears were made, air dried quickly and stained with May Grünwald-Giesma solution.

As illustrated in Fig. 3 the present myeloid leukaemia is similar to the myelomonocytic leukaemia (Naegeli) in man, showing indentation and lobulation of the nucleus and a comparatively large cytoplasm with occasional vacuoles and rather scant granules. This is especially prominent in the bone marrow, spleen or liver smears at the highly advanced leukaemic stage, although very often one can see typical, abnormal promyelo-, myeol-, or metamyelocytes in these organs as well as in the colonies.

*Spleen colony assay in the bone marrow*

Suspensions of leukaemic spleen cells from a mouse with advanced leukaemia were injected into 11 groups of mice, each animal being given about $10^5$ cells. Mice were killed daily from day 2 to day 12 and appropriate dilutions of bone marrow cell suspensions were injected into irradiated mice for assay. They were killed on the 9th day after injection for colony assay.

The number of colony-forming units per femur increased exponentially as a function of time between the 5th and 9th day of the leukaemic stage (Table III, Fig. 4). The colonies formed on day 2 and 4 are predominantly normal. After the 9th day the number of colony-forming units started to decline. This is probably due to cellular depletion which is commonly found in an advanced, disseminated leukaemic stage and results in extensive necrosis and fibrosis in the bone marrow.

The growth curve shown in Fig. 4 extrapolates at 2 hours time to about 5 CFU per femur. This value was experimentally confirmed by injecting appropriate dilutions of bone marrow cells into assay mice, 2 hours after injection of $1{\cdot}2 \times 10^5$ leukaemic cells into *irradiated* (900 rad.) donor mice.

Assuming one femur as about 5 per cent of the total haemopoietic space in the mouse, the "plating efficiency" of the injected leukaemic cells is about 1 : 1200 in the marrow. This calculation does not take into consideration

TABLE III.—*Number of Colony-forming Units Recoverable from the Femoral Marrow at Various Times after Intravenous Injection of* $1{\cdot}2 \times 10^5$ *Leukaemic Spleen Cell Suspension*

| Time after injection of leukaemic cells (days) | Cells per femur ($\times 10^7$) | No. of marrow cells injected into assay mice | Colonies per spleen (mean ± S.E.) | CFU per femur (mean ± S.E.) |
|---|---|---|---|---|
| 2 | 1·10 | $2 \times 10^5$ | 13·2 ± 1·19 | 726·0 ± 80·4 |
| 4 | 1·10 | $2{\cdot}5 \times 10^5$ | 17·8 ± 3·24 | 783·2 ± 136·6 |
| 5 | 0·88 | $1{\cdot}85 \times 10^5$ | 16·2 ± 2·58 | 770·0 ± 112·0 |
| 6 | 1·27 | $2 \times 10^4$ | 3·5 ± 0·30 | 2222·0 ± 211·6 |
| 7 | 1·12 | $2 \times 10^4$ | 10·5 ± 2·84 | 5880·0 ± 1190·1 |
| 8 | 1·08 | $4 \times 10^3$ | 6·0 ± 0·88 | 16200·0 ± 4563·8 |
| 9 | 1·90 | $4 \times 10^3$ | 8·2 ± 1·51 | 38950·0 ± 7570·2 |
| 10 | 0·79 | $2 \times 10^3$ | 3·4 ± 0·54 | 13331·2 ± 2468·7 |
| 11 | 0·91 | $2 \times 10^3$ | 4·2 ± 0·92 | 19211·1 ± 3774·7 |
| 12 | 0·76 | $1 \times 10^3$ | 1·4 ± 0·24 | 10640·0 ± 5687·3 |

FIG. 4.—Number of colony-forming units recoverable from the femoral marrow at various times after i.v. injection of $1 \cdot 2 \times 10^5$ spleen cell suspension.

possible " take " of injected leukaemic cells in other body sites, *e.g.* liver, or the possibility of preferential " take " in the spleen.

### DISCUSSION

In Table IV details of the myeloid leukaemia colony are compared with data from other cell studies. The AKR lymphoma (Bruce and van der Gaag, 1963) and leukaemia L1210 (Wodinsky, Swiniarski and Kensler, 1967) both form colonies in unirradiated syngeneic mice. In contrast to this the myeloid cells did not form colonies, although we have tried to produce the same result in unirradiated, syngeneic RFM/Un mice by injection of $10^2$ to $10^5$ leukaemic cells

TABLE IV.—*Comparison of Myeloid Leukaemia Colony with Other Already Described Types*

| Types of colony found in | Colonies first described by | Colonies formed in Irradiated mice | Colonies formed in Unirradiated mice | Details of cells seen in colonies |
|---|---|---|---|---|
| Normal, haemopoietic | Till and McCulloch (1961) | + | —* | Normal erythroid, myeloid and megakaryocytes. |
| AKR lymphoma | Bruce and van der Gaag (1963) | + | + | Immature lymphocytes. |
| Leukaemia L1210 (lymphocytic) | Wodinsky, Swiniarski and Kensler (1967) | + | + | . |
| Erythroleukaemia (erythroblastosis) | | | | |
| Friend type | Axelrad and Steeves (1964) | — | +† | Predominantly nucleated erythrocytes in different stages of differentiation. |
| Rauscher type | Pluznik and Sachs (1964) | — | +† | . |
| Myeloid leukaemia | Present report | + | — | Myeloid series; mainly abnormal promyelo-, myelo-, and metamyelocytes. |

* Except for unirradiated $WW^v$ mice, which will form colonies by injection with normal ww genotype mice (McCulloch, Siminovitch and Till, 1964).
† Endocolonies.

and by killing them 7 to 9 days after inoculation. Without irradiation, all leukaemic cells injected tended to infiltrate diffusely along the subcapsular area of the spleen as well as into the stroma. However, in irradiated assay mice the myeloid colonies showed very prominently as discrete colonies with compact cellularity. This demonstrates a histological as well as cytological difference from AKR lymphoma colonies seen in Fig. 5 and 6, which show prominent nucleoli, relatively scanty cytoplasm and, on section, a typical " starry sky " effect.

Lymphoma colonies have been found to develop in the kidneys of normal unirradiated mice (Bruce and van der Gaag, 1963). The kidney is one of the organs most frequently infiltrated by lymphoma or lymphocytic leukaemia. On the other hand, in murine myeloid leukaemia, the kidney is seldom infiltrated by leukaemic cells. This is also one of the major differences between the lymphoma group and myeloid leukaemia.

In the Friend or Rauscher type of colony, " focus " formation was found to occur in the spleen under the influence of cell-free extracts which had been filtered or heavily irradiated, but this did not occur if the animals had been irradiated supralethally (900 rad.) prior to injection of the extract (Axelrad and Steeves, 1964). Axelrad and Steeves concluded that foci represent new inductions which

EXPLANATION OF PLATES

FIG. 3.—Smear from a myeloid leukaemia colony. May-Giemsa. × 550.

FIG. 5.—Cross section of spleen showing myeloid leukaemia colonies 9 days after irradiation and injection of $2 \times 10^3$ leukaemic bone marrow cells. H. and E. × 14.

FIG. 6.—Smear from an AKR lymphoma colony. May-Giemsa. × 550.

FIG. 7.—An AKR lymphoma colony, at the 8th day after irradiation and injection of $10^3$ lymphoma cells. H. and E. × 140.

3

5

6

7

Tanaka and Lajtha.

result from the action of administered virus causing localised abnormal proliferation of host cells in the spleen. They also tested Gross' Passage A, Moloney and Graffi (known to be chloroleukaemia) viruses and found no spleen focus formation (Axelrad and Steeves, 1964). Therefore, these erythroleukaemia colonies are rather different from other lymphoma or myeloid leukaemia colonies in their origin.

A doubling time of 14·4 hours for the myeloid leukaemia compares with the doubling time of 11·2 hours in AKR lymphoma (Bruce and Meeker, 1964), and 9·5 hours (Wodinsky, Swiniarski and Kensler, 1967) and 13·2 hours (Skipper, Schabel and Wilcox, 1964) in leukaemia L1210. These times are significantly shorter than a doubling time of 20 to 25 hours observed in normal haemopoietic spleen colony-forming cells derived from transplanted marrow cells (McCulloch and Till, 1964).

Having established fundamental differences between lymphoma, erthroleukaemia and myeloid leukaemia colonies, one can apply the colony assay technique in uncertain reticuloendothelial malignancies for diagnostic purposes. Based on measurement of the survival fractions of myeloid leukaemia colony-forming units, sensitivity of transplanted myeloid leukaemia colony-forming cells to various chemotherapeutic agents is also feasible (Bruce, 1967).

### SUMMARY

Myeloid leukaemia cells in RFM/Un produced visible colonies in irradiated assay mice. A direct linearity was proved to exist between the number of colonies per spleen and cell inoculum. From leukaemic colony size at various days after inoculation, the doubling time for myeloid leukaemia colony cells in exponential phase was found to be approximately 14·4 hours.

Microscopic features of myeloid colonies were compared with other already described colony types, including normal haemopoietic, lymphoma and erythroleukaemia, by using mainly smear technique, and the morphological similarity of this murine myeloid leukaemia to myelomonocytic leukaemia (Naegeli) in man was emphasized.

T. Tanaka wishes to thank Dr. A. W. Craig, Department of Experimental Chemotherapy, Paterson Laboratories, for continuous encouragement and helpful advice.

### REFERENCES

Axelrad, A. A. and Steeves, R. A.—(1964) *Virology*, **24**, 513.
Bruce, W. R.—(1967) *Natn. Cancer Inst. Monogr.*, **24**, 249.
Bruce, W. R. and van der Gaag, H.—(1963) *Nature, Lond.*, **199**, 79.
Bruce, W. R. and Meeker, B. E.—(1964) *J. natn. Cancer Inst.*, **32**, 1145.
McCulloch, E. A. and Till, J. E.—(1964) *Radiat. Res.*, **22**, 383.
McCullock, E. A., Siminovitch, L. and Till, J. E.—(1964) Science, *N.Y.*, **144**, 844.
Pluznik, D. H. and Sachs, L.—(1964) *J. natn. Cancer Inst.*, **33**, 535.
Skipper, H. E., Schabel, F. M., Jr. and Wilcox, W. S.—(1964) *Cancer Chemother. Rep.*, **35**, 3.
Till, J. E. and McCulloch, E. A.—(1961) *Radiat. Res.*, **14**, 213.
Upton, A. C., Jenkins, V. K. and Conklin, J. W.—(1964) *Ann. N.Y. Acad. Sci.*, **114**, 189.
Wodinsky, I., Swiniarski, J. and Kensler, C. J.—(1967) *Cancer Chemother. Rep.*, **51**, 415.